# 2LS: Heap Analysis and Memory Safety (Competition Contribution)

**DOI:** 10.1007/978-3-030-45237-7_22

**Published:** 2020-03-13

**Authors:** Viktor Malík, Peter Schrammel, Tomáš Vojnar

**Affiliations:** 8 Johannes Kepler University, Linz, Austria; 9 University of Birmingham, Birmingham, UK; 10 Diffblue Ltd, Oxford, UK; 11 University of Sussex, Brighton, UK; 12 FIT, Brno University of Technology, Brno, Czech Republic

## Abstract

2LS is a framework for analysis of sequential C programs based on the CPROVER infrastructure and template-based synthesis techniques for checking both safety and termination. The paper presents the main improvements done in 2LS since 2018, which concern mainly the way 2LS handles dynamically allocated objects and structures as well as combinations of abstract domains.

### 1 Overview

2LS is a static analysis and verification tool for sequential C programs. At its core, it uses the $k$I$k$I algorithm ($k$-invariants and $k$-induction) [1], which integrates bounded model checking, $k$-induction, and abstract interpretation into a single, scalable framework. $k$I$k$I relies on incremental SAT solving in order to find proofs and refutations of assertions, as well as to perform termination analysis [2].

The 2019 and 2020 competition versions of 2LS feature product and power abstract domain combinations supporting invariant inference for programs manipulating shape and content of dynamic data structures [4]. Moreover, the 2020 version came with further enhancements for handling advanced features of memory allocation and made a step towards a support of generic abstract domain combinations.

**Architecture.** The architecture of 2LS has been described in previous competition contributions [7,5]. In brief, 2LS is built upon the CPROVER infrastructure [3] and thus uses *GOTO programs* as the internal program representation. The analysed program is translated into an acyclic, over-approximate single static assignment (SSA) form, in which loops are cut at the edges returning to the loop head. Subsequently, 2LS refines this over-approximation by computing inductive invariants in various abstract domains represented by parametrised logical formulae, so-called templates [1]. The competition version uses the zones domain for numerical variables combined with our shape domain for pointer-typed variables. The SSA form is bit-blasted into a propositional formula and given to a SAT solver. The $k$I$k$I algorithm then incrementally amends the formula to perform loop unwindings and invariant inference based on template-based synthesis [1].

* The Czech authors were supported by the project 20-07487S of the Czech Science Foundation.
** Jury member: imalik@fit.vut.cz.

## 2 New Features

The major improvements of 2LS since 2018 are mostly related to analysis of heap-manipulating programs. We build on the shape domain presented in 2018 [5] and introduce abstract domain combinations that allow us to analyse both shape and content of dynamic data structures. Furthermore, we introduce a special handling for the case when an address of a freed heap object is re-used for the next allocation.

Apart from an improved verification of heap-manipulating programs, we also introduce a generic skeleton of an abstract domain join algorithm, which is a step towards a support of generic abstract domain combinations.

### 2.1 Combinations of Abstract Domains

The capability of 2LS to jointly analyse shape and content of dynamic data structures takes advantage of the template-based synthesis engine of 2LS. Invariants are computed in various abstract domains where each domain has the form of a template while relying on the analysis engine to handle the domain combinators.

**Memory model** In our memory model, we represent dynamically allocated objects by so-called *abstract dynamic objects*. Each such object is an abstraction of a number of concrete dynamic objects allocated by the same malloc call [4].

**Shape Domain** For analysing the shape of the heap, we use an improved version of the shape domain that we introduced in 2018 [5]. The domain over-approximates the *points-to* relation between pointers and symbolic addresses of memory objects in the analysed program: for each pointer-typed variable and each pointer-typed field of an abstract dynamic object $p$, we compute the set of all addresses that $p$ may point to [4].

**Template Polyhedra Domain** For analysing numerical values, we use the template polyhedra abstract domains, particularly the *interval* and the *zones* domains [1].

**Shape and Polyhedra Domain Combination** Since both domains have the form of a template formula, we simply use them side-by-side in a product domain combination—the resulting formula is a conjunction of the two template formulae [4]. This combination allows 2LS to infer, e.g., invariants describing an unbounded singly-linked list whose nodes contain values between 1 and 10. We show an example of such a list in Figure 1. Here, all list nodes are abstracted by a single abstract dynamic object $ao_1$ (i.e. we assume that they are all allocated at the same program location). The invariant inferred by 2LS for such a list might look as follows:

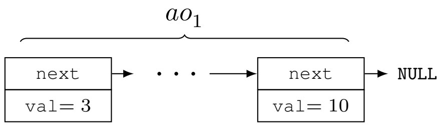

**Figure 1.** Unbounded singly-linked list abstracted by an abstract dynamic object $ao_1$.

$$(ao_1.next = \&ao_1 \vee ao_1.next = \texttt{NULL}) \wedge ao_1.val \in [1, 10].$$

The first disjunction describes the shape of the list—the *next* field of each node points to some node of the list or to `NULL`[1]. The second part of the conjunct is then an invariant in the interval domain over all values stored in the list—it expresses the fact that the value of each node lies in the interval between 1 and 10.

#### 2.2 Symbolic Paths

To improve precision of the analysis, we let 2LS compute different invariants for different *symbolic paths* taken by the analysed program. We require a symbolic path to express which loops were executed at least once. This allows us to distinguish situations when an abstract dynamic object does not represent any really allocated object and hence the invariant for such abstract dynamic object is not valid [4].

The symbolic path domain allows us to iteratively compute a set of symbolic paths $p_1, \ldots, p_n$ (represented by guard variables in the SSA) with associated shape and data invariants $I_1, \ldots, I_n$. The aggregated invariant is then $p_1 \Rightarrow I_1 \wedge \cdots \wedge p_n \Rightarrow I_n$, which corresponds to a power domain combination.

#### 2.3 Re-using Freed Memory Object for Next Allocations

In C, it is possible that, after a free is called, the freed memory is subsequently re-used when a malloc is called afterwards. Due to this, it may happen that the error state in the program in Figure 2 is reachable. This situation is, however, difficult to handle for 2LS as its memory model creates a unique abstract dynamic object for each malloc call. To overcome this limitation, we have introduced a special variable *fr* that is non-deterministically set to the value of the freed pointer at each free call. If two pointers $x$, $y$ are compared in the analysed program using a relational operator *op*, we transform the comparison $x \ op \ y$ into

```c
int *a = malloc(sizeof(int));
free(a);
int *b = malloc(sizeof(int));
if (a == b)
  // error state
```

**Figure 2.** Re-using a freed object

$$(x \ op \ y) \leftrightarrow ((x \neq fr \vee nondet_x) \wedge (y \neq fr \vee nondet_y)). \quad (1)$$

Here, $nondet_x$ and $nondet_y$ are unconstrained boolean variables modelling a non-deterministic choice. If neither $x$ nor $y$ has been freed, then the result of Eq. (1) is equal to $x \ op \ y$, but if either of the pointers might have been freed, then the result of Eq. (1) is non-deterministic, which makes our analysis sound for the described case.

#### 2.4 Generic Abstract Domain Templates

As is mentioned in Section 1, abstract domains are represented in 2LS by so-called templates. The main reason of templates is that they reduce the second-order problem of finding an inductive invariant to a first-order problem of finding values of template parameters. Apart from defining the form of the template (a parametrised logical formula), each abstract domain also needs to specify an algorithm to perform join of the current

[1] Here, $ao_1.f$ is an abstraction of the $f$ fields of all concrete objects represented by $ao_1$. Analogously, $\&ao_1$ is an abstraction of symbolic addresses of all represented objects.

values of template parameters with a model of satisfiability returned by an SMT solver. However, most of the domains use a similar approach to this algorithm, and therefore adding a new abstract domain to 2LS requires one to write an algorithm whose skeleton has already been written in existing domains.

To overcome this problem, we proposed a generic algorithm suitable for all existing abstract domains (see [6] for details). The main idea is based on the fact that most of the templates are conjunctions of multiple formulae, where each has its own parameter and describes a part of the analysed program, e.g., properties of a single program variable.

While this extension did not bring any additional functionality that would increase the score of 2LS in this year's edition of SV-COMP, it opened up possibilities for future enhancements, in particular (1) it simplifies adding of new abstract domains capable of analysing program properties that 2LS is currently not able to handle and (2) it is a significant step towards a support of generic abstract domain combinations that would allow 2LS to arbitrarily combine abstract domains and therefore analyse complex properties of programs requiring simultaneous reasoning in multiple domains.

### 3 Strengths and Weaknesses

One of the main strengths of 2LS is verification of programs requiring joint reasoning about shape and content of dynamic data structures. In 2019, we contributed 10 benchmarks into the ReachSafety category requiring such reasoning. The domain combination described in Section 2.1 allows 2LS to successfully verify 9 out of 10 of these benchmarks (the last one has timed out), making it the only tool capable of this apart from the category winner. Also, 2LS is notably strong in analysing termination, which is supported by the third place in the Termination category.

Still, there remain a lot of challenges and limitations. The main problem is that 2LS still lacks reasoning about array contents, and that it does not yet support recursion.

### 4 Tool Setup

The competition submission is based on 2LS version 0.8.[2] The archive contains the binaries needed to run 2LS (2ls-binary, goto-cc), and so no further installation is needed. There is also a wrapper script 2ls which is used by Benchexec to run the tools over the verification benchmarks. See the wrapper script also for the relevant command line options given to 2LS. The further information about the contents of the archive could be find in the README file. The tool info module for 2LS is called two_ls.py and the benchmark definition file 2ls.xml. As a back end, the competition submission of 2LS uses Glucose 4.0. 2LS competes in all categories except Concurrency and Java.

### 5 Software Project

2LS is implemented in C++ and it is maintained by Peter Schrammel with contributions by the community.[3] It is publicly available at http://www.github.com/diffblue/2ls under a BSD-style license.

[2] Executable available at https://doi.org/10.5281/zenodo.3678347.

[3] https://github.com/diffblue/2ls/graphs/contributors
